# Morphometric Study on the Mandibular Foramen

**DOI:** 10.7759/cureus.70862

**Published:** 2024-10-04

**Authors:** Sahasyaa Adalarasan, Lavanya Devi, Sathvik PS

**Affiliations:** 1 Medicine, Madras Medical College, Chennai, IND; 2 Anatomy, Institute of Anatomy, Madras Medical College, Chennai, IND

**Keywords:** human osteology, inferior alveolar nerve block (ianb), mandible, morphology, osteology

## Abstract

Aims and objectives

This study aimed to analyze the mandibular foramen (MF) by identifying its average vertical and horizontal diameters, determining its localization, and assessing the prevalence of accessory mandibular foramina.

Methodology

The study was conducted at the Institute of Anatomy, Madras Medical College, Chennai, India over one month in August 2024. A total of 77 human dry mandibles were examined, excluding those that were damaged to the extent that measurements could not be taken. The dimensions of the MF were measured in two directions: superoinferior and anteroposterior. Localization measurements were taken from the MF to key anatomical landmarks, including the coronoid notch, the inferior border of the ramus, the pterygomandibular raphe (third molar tooth), and the posterior border of the ramus. Measurements were recorded using digital vernier calipers and documented in Google Sheets (Google Inc., Mountain View, CA). The study also recorded the presence or absence of accessory MF.

Results

The superoinferior diameter of the MF was found to be 4.20 ± 0.94 mm (right (R)) and 4.28 ± 1.21 mm (left (L)). The anteroposterior diameter was found to be 3.55 ± 0.90 mm (R) and 3.60 ± 0.80 mm (L).

The distance between the MF and the coronoid notch was found to be 17.60 ± 2.95 mm (R) and 17.93 ± 3.16 mm (L). The distance between the MF and the inferior border was found to be 24.61 ± 4.32 mm (R) and 24.20 ± 4.28 mm (L). The distance between the MF and the pterygomandibular raphe was found to be 20.93 ± 1.98 mm (R) and 20.63 ± 2.00 mm (L). The distance between the MF and the posterior border was found to be 11.31 ± 1.67 mm (R) and 11.70 ± 2.28 mm (L).

This present study identified 20 instances of an accessory MF, with 10 occurring on each side. Additionally, 10 mandibles displayed accessory mandibular foramina on both sides.

Conclusions

The comprehensive analysis of the MF and its anatomical landmarks in this study offers valuable insights that enhance our understanding of mandibular anatomy, with direct implications for clinical practice. The identification of the pterygomandibular raphe as a measurable landmark from within the buccal cavity introduces a practical guide for dental practitioners and maxillofacial surgeons, potentially improving the accuracy of surgical and anesthetic procedures. Furthermore, the detailed measurements of MF width provide a foundation for refining anesthetic techniques, thereby enhancing patient outcomes. The observed prevalence of accessory MF underscores the importance of considering anatomical variability in clinical practice, highlighting the need for continuous anatomical research to advance clinical methodologies and ultimately improve patient care and surgical success.

## Introduction

The mandibular foramen (MF) is an anatomical structure situated on the medial surface of the ramus of the mandible. 

This opening facilitates the passage of the inferior alveolar nerve and vessels. The inferior alveolar nerve, a branch of the mandibular nerve (V3), innervates the lower dentition, lower lip, and a portion of the chin. 

The MF is a critical landmark in dental procedures, particularly for the administration of local anesthetics during dental procedures [1]. Misestimations of its location can contribute to unsuccessful anesthesia of the inferior alveolar nerve, with failure rates said to be approximately 20%-25% [2]. Removal of the impacted third molar is one of the most common uses of this procedure. The success of this technique is contingent upon the precise positioning of the anesthetic needle in relation to the MF. Accurate localization is essential during osteotomies, such as sagittal split ramus osteotomy (SSRO) and intraoral vertical ramus osteotomy (IVRO), where precise knowledge of the MF’s location can mitigate the risk of undesirable mandibular fractures [3]. 

This study aims to observe the morphology and morphometry in detail, utilizing skeletal samples to enhance the understanding of the MF. The findings are expected to inform more effective clinical practices and improve the efficacy of surgical interventions in the mandibular region. Ultimately, this research seeks to provide valuable insights for personalized and precise medical care, acknowledging the unique anatomical variations across diverse mandibular samples.

## Materials and methods

The proposal for this study was submitted to the Institutional Ethics Committee at Madras Medical College, Chennai, India, and received approval before the commencement of the research (approval number: 52072024). The study was conducted at the Institute of Anatomy, Madras Medical College, over the span of one month during August 2024. A total of 77 human dry mandibles were selected for analysis. These mandibles were carefully chosen to exclude any that were damaged or otherwise compromised, ensuring that only those suitable for precise measurement were included in the study.

Each mandible underwent a thorough inspection to confirm its integrity. Following inspection, the mandibles were meticulously cleaned and dried to remove any residual organic material or dust that could interfere with accurate measurements. This step was crucial to ensure that all surfaces were free of contaminants, thereby improving the precision of the measurements.

For the measurement process, a high-precision digital vernier caliper was employed, with an accuracy of 0.01 mm, to obtain the most accurate data possible. Before each use, the caliper was calibrated to maintain consistency throughout the study. The dimensions of the MF were specifically measured in two directions, superoinferior and anteroposterior, using the smaller jaws of the vernier caliper. The caliper was carefully positioned to ensure that the measurements were free from parallax errors, and three readings were taken for each dimension, with the average being recorded to account for any slight variations.

In addition to measuring the dimensions of the MF, the study also aimed to localize the MF in relation to key anatomical landmarks. Measurements were taken from the MF to the coronoid notch, the inferior border of the ramus, the pterygomandibular raphe (aligned with the third molar tooth), and the posterior border of the ramus. For these longer distances, the larger jaws of the vernier caliper were utilized to ensure stability and maintain measurement accuracy. Each measurement was repeated three times, and the average was documented to minimize any potential discrepancies.

Beyond the primary measurements, the study also included an assessment of the presence or absence of accessory mandibular foramina. Each mandible was carefully examined to identify any additional foramina, and their locations were recorded meticulously. This aspect of the study was particularly important as it provided additional data on anatomical variability, which is critical for clinical applications.

All measurements and observations were initially recorded manually on pre-designed data collection sheets. These records were then reviewed for accuracy before being transferred to the Google Sheets application (Google Inc., Mountain View, CA). The digital format facilitated the organization of data and enabled the performance of various statistical analyses. Key statistical measures, including mean values, standard deviations, and comparative analyses between the right and left sides, were calculated using the built-in functions of Google Sheets. These statistical analyses were essential for interpreting the data and drawing meaningful conclusions from the study.

## Results

The superoinferior diameter of the MF was found to be 4.20 ± 0.94 mm (right (R)) and 4.28 ± 1.21 mm (left (L)) (Table 1). The anteroposterior diameter was found to be 3.55 ± 0.90 mm (R) and 3.60 ± 0.80 mm (L) (Table 2).

**Table 1:** Superoinferior diameter of the mandibular foramen

Side	Minimum (in mm)	Maximum (in mm)	Mean (in mm)	SD (in mm)
Right	1.60	6.00	4.19	0.94
Left	2.10	6.00	4.28	1.21

**Table 2:** Anteroposterior diameter of the mandibular foramen

Side	Minimum (in mm)	Maximum (in mm)	Mean (in mm)	SD (in mm)
Right	1.80	5.90	3.55	0.89
Left	2.20	4.80	3.60	0.80

The distance between the MF and the coronoid notch was found to be 17.60 ± 2.95 mm (R) and 17.93 ± 3.16 mm (L) (Table 3). The distance between the MF and the inferior border was found to be 24.61 ± 4.32 mm (R) and 24.20 ± 4.28 mm (L) (Table 4). The distance between the MF and the pterygomandibular raphe was found to be 20.93 ± 1.98 mm (R) and 20.63 ± 2.00 mm (L) (Table 5). The distance between the MF and the posterior border was found to be 11.31 ± 1.67 mm (R) and 11.70 ± 2.28 mm (L) (Table 6).

**Table 3:** Distance between the mandibular foramen and the coronoid notch

Side	Minimum (in mm)	Maximum (in mm)	Mean (in mm)	SD (in mm)
Right	10.10	24.30	17.60	2.95
Left	10.30	27.70	17.93	3.16

**Table 4:** Distance between the mandibular foramen and the inferior border

Side	Minimum (in mm)	Maximum (in mm)	Mean (in mm)	SD (in mm)
Right	15.40	37.60	24.61	4.32
Left	14.20	33.80	24.20	4.28

**Table 5:** Distance between the mandibular foramen and the pterygomandibular raphe

Side	Minimum (in mm)	Maximum (in mm)	Mean (in mm)	SD (in mm)
Right	14.30	29.30	20.93	1.98
Left	14.60	29.70	20.65	1.99

**Table 6:** Distance between the mandibular foramen and the posterior border

Side	Minimum (in mm)	Maximum (in mm)	Mean (in mm)	SD (in mm)
Right	7.50	14.20	11.31	1.67
Left	7.90	19.60	11.70	2.28

This present study identified 20 instances of an accessory MF, with 10 occurring on each side. Additionally, 10 mandibles displayed accessory mandibular foramina on both sides (Table 7).

**Table 7:** Prevalence of the accessory mandibular foramen

Side	Number	Percentage (%) of the sample size
Left (exclusively)	10	6.49
Right (exclusively)	10	6.49
Bilaterally	10	6.49
Total	40	25.97

## Discussion

The present study's measurements of the distances from the MF to the anatomical landmarks are consistent with previous studies done across India and the world [1-6]. However, these distances lack external accessibility, limiting their clinical utility. This aligns with the consensus in the field that while these internal anatomical markers are reliable, their practical application in clinical settings is limited due to their inaccessibility during routine dental procedures.

A significant finding in the present study is the distance between the pterygomandibular raphe to an MF, an anatomical landmark that can be approached through the buccal cavity. This distance is measured at approximately 20.78 ± 1.98 mm, representing a valuable measurement for dental practitioners and maxillofacial surgeons. The buccal accessibility of this landmark offers a practical guide for dentists and oral surgeons, enhancing the precision of interventions involving the mandibular region.

Moreover, the present study introduces the measurement of the anteroposterior diameter of the MF as a potential metric for improving anesthetic tools used in dental procedures. The precise measurement of MF width could lead to the development of more effective and targeted anesthetic techniques, thereby improving patient outcomes and procedural efficiency.

The present study also highlights the presence of the accessory MF, which is known to contain a branch/tributary of the inferior alveolar vessels. We found the accessory MF in 25.97% of all rami of the mandibles examined, a lower prevalence than reported by previous studies by 14.93% [7]. This discrepancy may be attributed to the larger sample size in the present study, which enhances the statistical power and reliability of its findings. The presence of the accessory MF has significant implications for surgical planning and anesthetic management, as it represents an additional anatomical consideration for clinicians [8].

The position of the MF has significant implications for the success of inferior alveolar nerve blocks. Studies utilizing cone-beam computed tomography (CBCT) have provided detailed anatomical insights that aid in the localization of the MF for effective anesthesia delivery [9, 10]. Accurate localization is essential to avoid complications and ensure adequate anesthesia during dental procedures.

Research has also highlighted the importance of alternative techniques such as the Vazirani-Akinosi and Gow-Gates nerve blocks, which may offer advantages over traditional methods in specific clinical scenarios [11]. These techniques rely on different anatomical landmarks, emphasizing the need for a comprehensive understanding of mandibular anatomy. Furthermore, studies have explored the variability in the position of the mandibular lingula, an important reference point for nerve blocks. The variability underscores the necessity for individualized approaches based on detailed anatomical assessments.

Reitzik et al. (1976) provided foundational insights into the MF's positioning, highlighting its relevance for the effective administration of local anesthesia [12]. Subsequent research has underscored the variability in MF location across different populations and age groups, as observed by Lopes et al. (2010), who detailed the morphological variations in mandibles of varying demographics [13]. The identification of accessory mandibular foramina further complicates surgical planning, with Murlimanju et al. (2014) noting its prevalence and the potential embryological basis for its occurrence [14]. In an Indian population, Das et al. (2010) explored these anatomical anomalies, emphasizing the need for clinicians to consider such variations during interventions involving the mandible [15].

## Conclusions

The present study's comprehensive analysis of the MF and associated anatomical landmarks provides crucial insights that enhance our understanding of mandibular anatomy and its clinical applications. The pterygomandibular raphe, measurable via the buccal cavity, introduces a practical landmark for dental practitioners and maxillofacial surgeons, potentially improving the precision of surgical and anesthetic procedures. Additionally, the identification of the MF width offers new avenues for refining anesthetic techniques, thus enhancing patient outcomes. The higher prevalence of the accessory MF observed in the present study underscores the importance of considering anatomical variability in clinical practice.

These findings not only corroborate previous research but also expand the scope of anatomical knowledge with clinically relevant landmarks. By integrating these novel measurements into clinical practice, dental professionals can achieve greater accuracy and effectiveness in procedures involving the mandibular region. This study underscores the continuous need for anatomical research to inform and advance clinical methodologies, ultimately contributing to improved patient care and surgical success.
